# Signal Transducers and Activators of Transcription-1 (STAT1) Regulates microRNA Transcription in Interferon γ-Stimulated HeLa Cells

**DOI:** 10.1371/journal.pone.0011794

**Published:** 2010-07-26

**Authors:** Guohua Wang, Yadong Wang, Mingxiang Teng, Denan Zhang, Lang Li, Yunlong Liu

**Affiliations:** 1 School of Computer Science and Technology, Harbin Institute of Technology, Harbin, Heilongjiang, People's Republic of China; 2 Department of Medical and Molecular Genetics, Indiana University School of Medicine, Indianapolis, Indiana, United States of America; 3 Division of Biostatistics, Department of Medicine, Indiana University School of Medicine, Indianapolis, Indiana, United States of America; 4 Center for Computational Biology and Bioinformatics, Indiana University School of Medicine, Indianapolis, Indiana, United States of America; 5 Center for Medical Genomics, Indiana University School of Medicine, Indianapolis, Indiana, United States of America; University of Calgary, Canada

## Abstract

**Background:**

Constructing and modeling the gene regulatory network is one of the central themes of systems biology. With the growing understanding of the mechanism of microRNA biogenesis and its biological function, establishing a microRNA-mediated gene regulatory network is not only desirable but also achievable.

**Methodology:**

In this study, we propose a bioinformatics strategy to construct the microRNA-mediated regulatory network using genome-wide binding patterns of transcription factor(s) and RNA polymerase II (RPol II), derived using chromatin immunoprecipitation following next generation sequencing (ChIP-seq) technology. Our strategy includes three key steps, identification of transcription start sites and promoter regions of primary microRNA transcripts using RPol II binding patterns, selection of cooperating transcription factors that collaboratively function with the transcription factors targeted by ChIP-seq assay, and construction of the network that contains regulatory cascades of both transcription factors and microRNAs.

**Principal Findings:**

Using CAMDA (Critical Assessment of Massive Data Analysis) 2009 data set that includes ChIP-seq data on RPol II and STAT1 (signal transducers and activators of transcription 1) in HeLa S3 cells in control condition and with interferon γ stimulation, we first identified promoter regions of 83 microRNAs in HeLa cells. We then identified two potential STAT1 collaborating factors, AP-1 and C/EBP (CCAAT enhancer-binding proteins), and further established eight feedback network elements that may regulate cellular response during interferon γ stimulation.

**Conclusions:**

This study offers a bioinformatics strategy to provide testable hypotheses on the mechanisms of microRNA-mediated transcriptional regulation, based upon genome-wide protein-DNA interaction data derived from ChIP-seq experiments.

## Introduction

MicroRNAs are small non-coding RNAs known to regulate the target transcripts by promoting mRNA degradation and suppressing translation [1], [2]. To date, several hundred precursor microRNAs (pre-microRNAs) and mature microRNAs have been annotated in several mammalian genomes [3]. Investigating the microRNA-mediate regulatory network is important in understanding transcriptional and post-transcriptional regulatory mechanisms through which cells respond to certain biological stimulation. During the past decades, it has been reported in several studies that transcription factors and microRNAs form regulatory circuits, where transcription factors regulate microRNA transcription, which in turn controls the expression levels of transcription factors post-transcriptionally [4], [5], [6], [7]. Several studies also reported strategies in identifying core promoters of microRNAs through sequence features [8], [9] or chromatin marks [10].

A combination of high throughput technologies and bioinformatics analysis provides important means in generating a testable hypothesis for mechanistic study. In recent years, chromatin immunoprecipitation following next generation sequencing technology (or ChIP-seq) has been widely used to profile the binding patterns of DNA binding proteins in a genome-wide scale, including transcription factors [11], [12], [13], histone marks [14], [15], and RNA polymerase II [11], [14], [16]. These provide unprecedented information in understanding complicated mechanisms of regulating gene expression. In this study, we report a bioinformatics strategy in identifying the microRNA-mediate regulatory network from ChIP-seq-dervied genome-wide binding data on RPol II and transcription factor(s). We applied this strategy to the ChIP-seq data provided in the CAMDA (Critical Assessment of Massive Data Analysis) 2009 challenging data set [11], which includes ChIP-seq data on RPol II in HeLa S3 cells, and ChIP-seq data on a transcription factor STAT1 (signal transducers and activators of transcription-1) in the same cell line with interferon γ stimulation. Interferon γ is a cytokine that is critical for cellular immune response against bacterial and viral infections [17]. Cellular response to interferon γ is known to activate the JAK (Janus family kinase)-STAT signaling pathway to control transcription of target genes via specific response elements [18]. Several recent studies reported that microRNAs are involved in the interferon-induced cellular response through targeting STAT1 and/or its co-factors [19], [20], [21]. In this study, we design a bioinformatics strategy that systematically predicts such relationship based on high throughput functional genomics data. Using RPol II ChIP-seq data, we identified active promoter regions of 83 microRNAs in HeLa cells, of which, 41 were directly targeted by the STAT1 binding sites. We have also identified 8 potential feedback relationships involving STAT1-targeting microRNAs, interferon γ gene, STAT1, or/and AP-1 binding proteins, together with many potential incoherent feed-forward loops. This strategy provides important testable hypotheses for further mechanistic understanding of cellular immune response to interferon γ treatment.

## Results

In order to understand the role of the microRNA-mediate regulatory network in interferon γ – stimulated HeLa cells, we conducted bioinformatics analysis of the RPol II and STAT1 ChIP-seq data provided by the CAMDA 2009 challenging data set [11]. Our analysis includes three major steps: (1) determining promoter regulatory regions for intergenic microRNAs in HeLa cells; (2) identifying transcriptional co-factor(s) that jointly work with STAT1; and (3) constructing potential regulatory network motifs that are involved in microRNA-mediated cell response.

### Determining promoter regulatory regions for intergenic microRNAs

**Figure 1:**
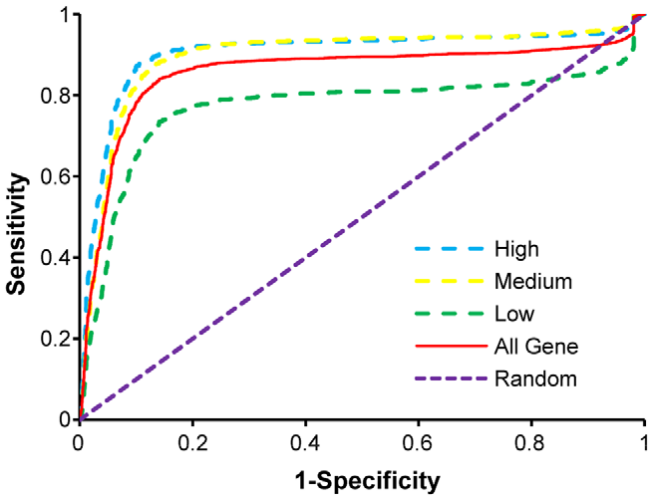
ROC curve for TSS prediction of protein coding genes. The expressed genes were separated into three categories, high (light blue), low (green), and medium expressed genes (yellow). The three categories of expressed genes and non-expressed genes are considered positive and negative sets, respectively. One fourth of the genes are used as training data, while the remaining are used as the test set. The ROC curve was generated using ROCR library in R project (http://www.r-project.org).

In this study, we indentified promoter regions of intergenic microRNAs in HeLa cells using the RPol II ChIP-seq data provided by the CAMDA 2009 challenging data set [11]. By assuming that RPol II binding distribution around the transcription start sites (TSS) is similar for microRNAs and protein coding genes, our workflow includes three components [22], 1) modeling binding patterns of RPol II around TSS of highly expressed protein-coding genes, 2) evaluating performance of the model, and 3) predicting promoter regions upstream of annotated microRNAs using the inferred model. In the first step, highly expressed genes were selected based upon microarray experiment using Affymetrix platform (GEO number: GSE3051 [23]). Following a strategy similar to what we did previously [22], we focused only on the genes whose transcript lengths are greater than 10,000-bp and no other genes are present within 10,000-bp of their TSS. This analysis resulted in identification of 4,120 expressed genes and 2,682 unexpressed genes in HeLa cells, based on the absent and present calls using the Affymetrix Microarray Suite®, version 5.0 [24]. To evaluate the predictive power of our model to identify active promoters in HeLa cells using provided RPol II ChIP-seq data, we randomly selected 1/4 of expressed genes to train our model. The remaining genes, both expressed and non-expressed, were used as test sets. The area under the curve (AUC) in the Receiver Operator Characteristic (ROC) reached 0.86 in differentiating all the expressed genes and unexpressed genes (Figure 1), suggesting the excellent predictive power of our strategy. We further divided the expressed gene into three categories based on their expression levels (low expression, medium expression and high expression), of which each category contains the same number of genes. The result of this analysis (Figure 1) clearly demonstrates that the prediction accuracy of our model is higher for the genes that are highly expressed.

**Figure 2:**
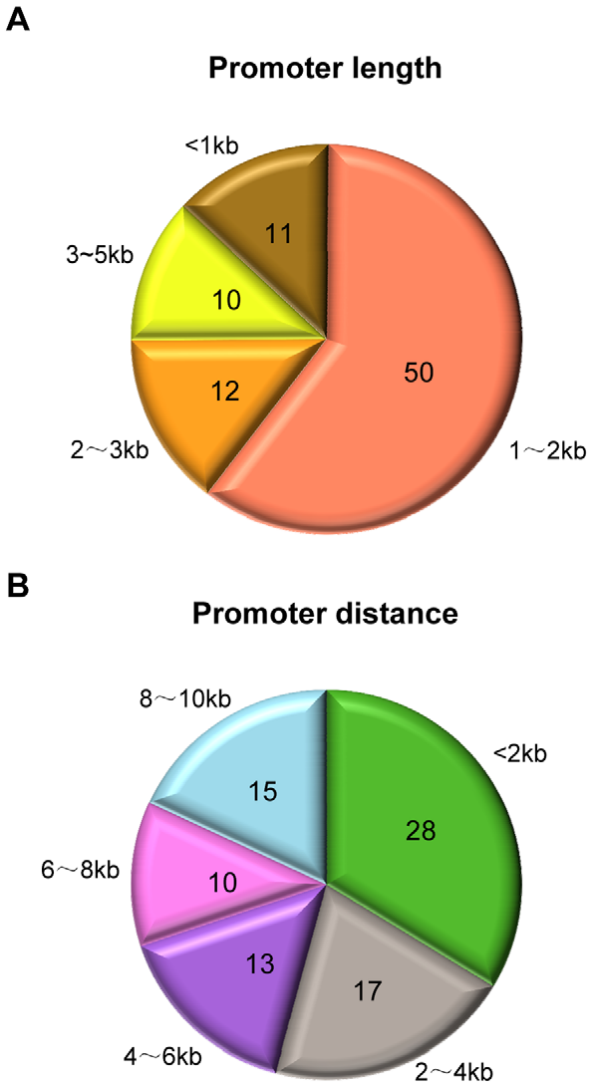
Statistics of predicted microRNA promoters. Pie diagram shows the numbers of microRNAs with different ranges of (A) promoter lengths and (B) distances between their predicted transcription start sites and annotated mature and pre-microRNAs.

We obtained annotations of 685 human mature or pre-microRNAs from the miRBase microRNA sequence database (version 11.0, [3]). Among them, 419 intergenic microRNAs (located between protein-coding genes) were used for promoter identification. Using the model parameters estimated based on RPol II binding patterns around the transcription start sites of protein coding genes, we indentified 83 active microRNA promoters in HeLa cells (with false discovery rate ≤0.2, Supplementary Table S1). The median length of regulatory region was 1,476-bp, with longest and shortest widths of 4,989-bp and 397-bp, respectively (Figure 2A). These regions are believed to be around transcription start sites of pri-microRNA, which may be hundreds or thousands of nucleotides in length and contain one or more microRNA stem loops; pri-microRNAs are further processed to pre- and then to mature microRNA forms [25], [26]. The distances between the identified TSS and their corresponding mature or pre-microRNA also differ in a great deal, ranging from 200 to 10,000-bp, with median distance around 3,600-bp (Figure 2B).

**Figure 3:**
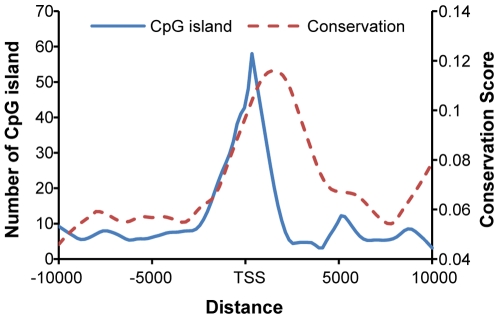
Sequence features around predicted microRNA promoters. CpG islands and conservation scores were retrieved from the UCSC genome browser, where CpG islands were defined as genomic regions with a length greater than 200 bp, with a minimal GC content of 50%, and a ratio of observed/expected CpG greater than 0.6. The conservation scores were calculated based on a phylogenetic hidden Markov model that measures the evolutionary conservation in 17 vertebrates [27].

We further examined the sequence features of identified promoter regions, including their conservation levels across evolution and their relationship with annotated CpG islands. We observed high GC content within or around the predicted regulatory regions. Among the 83 predicted microRNA promoters, 66 promoters (79.5%) were found to either contain or overlap with annotated CpG islands [27]. This result is highly significant, with P-value<10^−77^ ; this p-value is calculated based on 10,000-time permutation evaluating the possibility that 66 out of 83 randomly selected regions overlap or contain with CpG island. In addition, the identified promoter region and transcription start site also demonstrated higher conservation (PhastCons scores based on 17 species, including mammalian, amphibian, bird, and fish [27]), compared to randomly selected regions (red dash line in Figure 3).

### Identifying collaborating transcription factor(s) that bind to sites adjacent to the STAT1 binding region

Interferon stimulation may influence STAT1 binding by the following three mechanisms, (1) interferon γ treatment affects STAT1 binding affinity in HeLa cells, which will potentially change the genome-wide binding pattern of STAT1 protein; (2) interferon treatment recruits other transcription factors interacting with STAT1, which permits STAT1 association with DNA through protein-protein interaction; and (3) interferon γ changes the DNA binding activity of collaborating transcription factors that bind to sites adjacent to the STAT1 binding regions. To explore the possibilities of these potential mechanisms, we scanned ChIP-seq-derived STAT1 binding regions for enrichment of 741 biologically-validated transcription factor binding sites documented in the TRANSFAC database [28]. Position-specific score matrices (PSSM) were used to calculate the possibility of a specific transcription factor binding at one genomic locus, as described previously [29]. This analysis selects transcription factors whose binding sites are enriched in ChIP-enriched regions compared to background promoters (see Methods).

**Figure 4:**
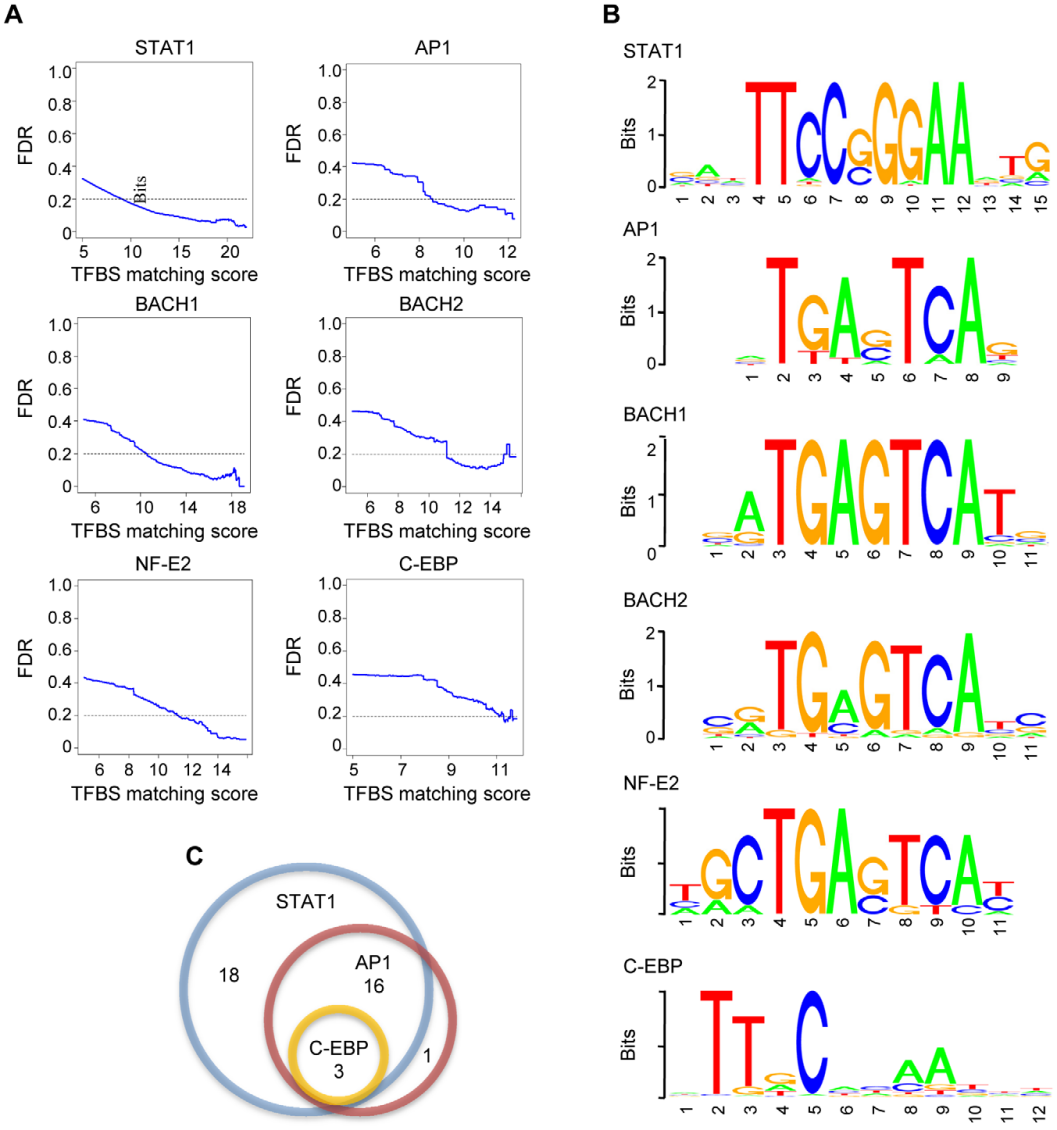
Identification of collaborating transcription factors. (A) Enriched motifs in STAT1 binding regions. The y axes demonstrate the ratio of the number of specific binding in randomly selected promoter sequences (1,000-bp upstream transcription start site of the genes that are not STAT1 targets) and the sequences in ChIP-enriched regions; this number can also be considered as false discovery rate (FDR) at different matching scores (represented by x axes). (B) Sequence logos of enriched motifs. The size of the letter indicates the information content of that nucleotide position. (C) Number of microRNAs whose predicted promoter regions overlap with STAT1-enriched regions that contain putative binding sites of STAT1, AP-1, and C/EBP.

Twenty-six PSSMs representing binding sites of 12 transcription factors are enriched in the STAT1 binding regions (Supplementary Table S2). Among these PSSMs, binding sites of 6 transcription factors can be found within the identified microRNA regulatory regions (Supplementary Table S3). A binding instance was defined if the matching score between its oligonucleotide sequence and the PSSM of the transcription factor binding sites is higher than a PSSM-specific cutoff, which was determined by the lowest matching score where the density of positively identified binding sites in the ChIP-enriched regions is 5 times more than the one in the background sequences (or FDR of ≤20%). Figure 4A demonstrates that for these 6 transcription factors, with the matching score cutoff increases, the enrichment of the binding sites in ChIP-selected regions also increases; the Y-axes in the figure denotes the ratio of the density of specific binding sites in randomly selected promoter regions to the ones in the ChIP-enriched regions, or false discover rate (FDR); a smaller FDR indicates higher enrichment.

Importantly, as expected, STAT1 binding sites are the predominant elements enriched in STAT1 binding regions. This serves as an important positive control that a combination of a ChIP-seq experiment and our computational strategy is capable of identifying the binding sites being targeted. In addition, the sequence logos of the 6 transcription factors are shown in Figure 4B. Clearly, the consensus motifs of BACH1, BACH2, and NF-E2 are very similar to the core sites of AP-1 binding motif. This may represent an artifact that the selection of these factors is due to their sequence similarity with AP-1 binding sites. In order to avoid this potential artifact, we focus our future analysis on binding sites of three transcription factors, STAT1, AP-1, and C/EBP (CCAAT-enhancer-binding proteins).

### Role of STAT1 and its cofactor in regulating microRNA transcription

We examined overlapping of ChIP-seq-derived STAT1 binding regions, identified by Gerstein's group [11], with 83 predicted microRNA promoters. Among these, promoter regions of 41 microRNAs (49.4%, Supplement Table S1) contain or overlap with ChIP-seq-derived STAT1 enriched regions; this finding is statistically significant when we repeated permutation analysis 10,000 times, and calculated the possibility that 41 out of 83 randomly-selected regions overlap with STAT1 binding regions (P-value <10^−39^). These represent the microRNAs that are potentially regulated by STAT1 in HeLa cells in response to interferon γ stimulation, following one of the 3 potential mechanisms (direct STAT1 binding, STAT1 collaborating with other transcription factor, or STAT1 interacting with DNA through protein-protein interaction). Most promoters contain one binding site, while the promoters of hsa-mir-21 and hsa-mir-92b have two STAT1 target sites, and a microRNA cluster, hsa-mir-193b and hsa-mir-365-1, has three target sites.

Among the 41 microRNAs whose identified promoter regions include or overlap with STAT1 enriched regions, 38 encompass ChIP-seq regions that contain one or more binding sites of STAT1 and/or its identified cofactors, AP-1, or C/EBP. Among these ChIP-seq regions, 37 such regions contain STAT1 binding sites (Supplementary Table S3). This suggests that under interferon γ treatment, STAT1 regulates microRNA transcription mainly through direct binding to their promoter regions. Interestingly, 19 out of 37 (or 51.4%) STAT1-containing regions also enclose AP-1 binding sites (Figure 4C). This indicates that AP-1 can potentially bind to the regions adjacent to the STAT1 binding sites, and serves as a collaborating factor in regulating microRNA transcription. To test the statistical significance of this finding, we randomly selected 37 regions from gene promoters and counted the number of regions that contain AP-1 binding sites. We repeated this practice 10,000 times, and then calculated the P-value for our finding is 0.0056, based upon binomial distribution. In addition, 3 regions containing both STAT1 and AP-1 binding sites also encompass C/EBP binding sites. This suggests that multiple transcription factors may be involved in the transcriptional machinery.

### Construct potential regulatory network motifs that are involved in microRNA-mediated cell response

Based upon the regulatory roles of STAT1 and its collaborating factors on microRNA transcription, we can derive the potential network elements that describe microRNA-mediated cell response. In this study, we are interested in two types of network motifs, the feedback and feed-forward loops, where the feedback relationship describes the roles STAT1-targeting microRNAs playing in regulating STAT1 and its collaborating transcription factors, and feed-forward relationship denoting their roles in regulating other STAT1-targeted genes.

**Figure 5:**
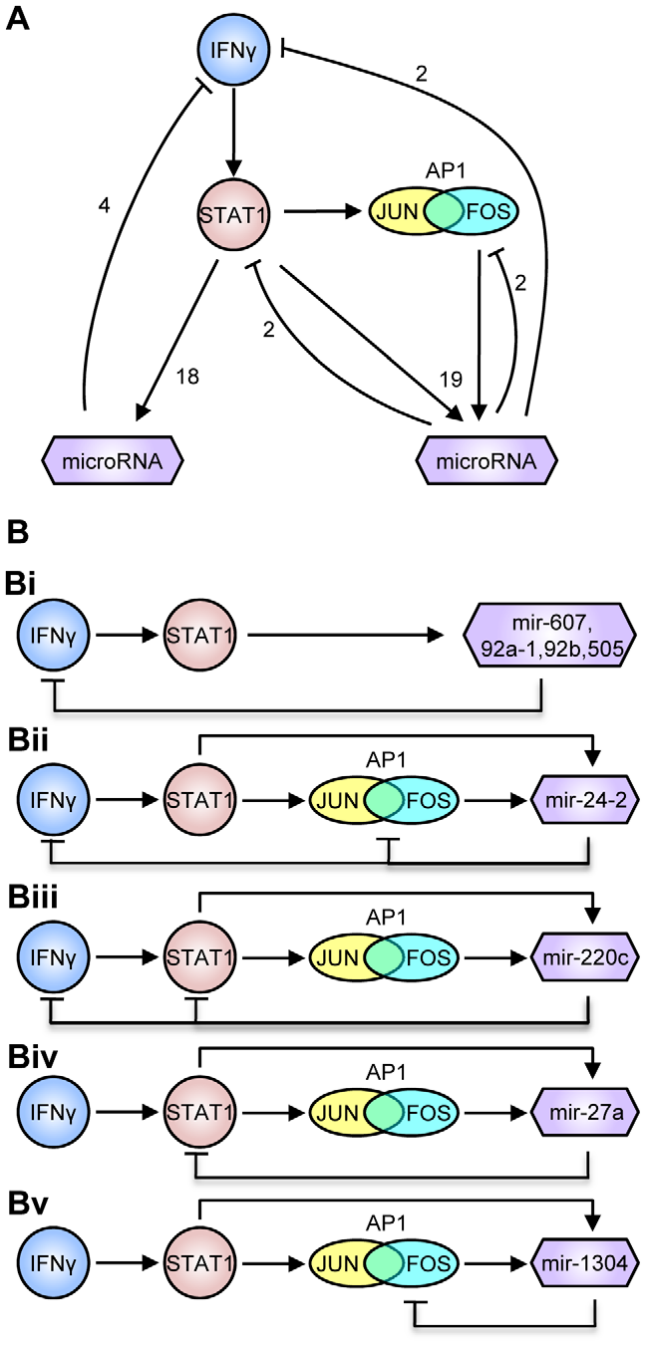
Feedback network motifs. (A) Overall representation of the feedback relationships. This network contains interferon γ, STAT1, two AP-1 binding proteins, JUN and FOS, and 37 microRNAs whose predicted promoter regions overlap with STAT1 ChIP-seq regions that contain putative STAT1 and/or AP-1 binding sites. (B) Five instances where STAT1-targeting microRNAs potentially target interferon γ, STAT1, JUN or FOS proteins.

**Feed-back loops** **.** The overall scheme of feedback regulation is shown in Figure 5A. This scheme includes two major components, one of which only involves STAT1 regulation, and the other one includes both STAT1 and its collaborating factor AP1. From ChIP-seq data, we have observed STAT1-enriched binding sites around the transcription start sites of JUN and FOS genes, two transcription factors that target AP-1 binding sites. Therefore, upon interferon γ stimulation, STAT1 may stimulate JUN and/or FOS, and both STAT1 and JUN/FOS collaboratively regulate microRNA expression.

Promoter regions of 4 microRNAs, miR-607, miR-92a-1, miR-92b, and miR-505, encompass or overlap with STAT1-enriched regions; they also potentially target the 3′-untranslated regions (3′-UTR) of interferon γ gene (Figure 5Bi). This may represent a feedback relationship that interferon effects are attenuated through the STAT1-microRNA-mediated network. Similar relationships also involve miR-1304, miR-24-2, miR-27a, and miR-220c. For instance, the predicted promoter regions of miR-24-2, miR-220c, miR-27a, and miR-1304 all overlap with a STAT1 ChIP-seq region, which includes both STAT1 and AP-1 binding sites. This suggests that these microRNAs are co-regulated by STAT1 and AP-1 binding proteins. Importantly, miR-24-2 can in turn target the 3′-UTRs of Interferon γ, JUN, and FOS genes (Figure 5Bii) and therefore potentially inhibit the mRNA expression levels of these genes. Similarly, miR-220c targets both STAT1 and interferon γ (Figure 5Biii); miR-27a targets STAT1 (Figure 5Biv), and miR-1304 targets FOS gene (Figure 5Bv). The feedback relationship may represent a microRNA-mediated molecular mechanism by which HeLa cells maintain homeostasis during interferon γ treatment.

**Figure 6:**
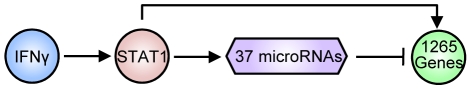
Potential feed-forward regulatory relationships. The feed-forward relationship is defined as the STAT1-targeting microRNAs also potentially regulating STAT1-targeting genes. This represents a potential incoherent relationship by which HeLa cells maintain fine-tune transcriptional control.

**Feed-forward relationship** **.** The feed-forward relationship denotes the roles of STAT1-regulated microRNAs on the post-transcriptional suppression of STAT1-targeting mRNAs. Since STAT1 can potentially exert both stimulatory and inhibitory roles on the target gene, it may represent a mechanism that HeLa cells fine tune the regulation of STAT1-induced activation. Such a relationship, also called “incoherent feed-forward regulation” [30], is also observed in Marson et al. [10]. Among 6,264 genes which STAT1-enriched ChIP-seq regions encompass or overlap with their regulatory regions (−1,000bp to +500bp from transcription start sites), 1,265 genes (20.2%) are also predicted targets of 37 STAT1-targeting microRNAs (Figure 6). A list of putative feed-forward relationships can be found in Supplementary Table S4.

## Discussion

We report a bioinformatics strategy to study the microRNA-mediated regulatory network, based on ChIP-seq-derived genome-wide binding patterns of transcription factors and RNA polymerase II. Our strategy includes three components, predicting microRNA promoter regions, identifying transcription cofactors, and deriving key microRNA-mediated regulatory network elements. We applied this strategy to the 2009 CAMDA challenging data set [11], in which genome-wide binding patterns of RPol II and STAT1 were measured in HeLa S3 cells under control and interferon γ stimulated conditions, respectively. We identified promoter regions of 83 microRNAs, 41 of which were directly targeted by STAT1 upon interferon γ stimulation. We have also identified AP-1 and C/EBP as collaborating transcription factors, whose binding sites are enriched in ChIP-seq-derived STAT1 binding regions. By integrating the results from microRNA target prediction, we derived several putative feedback and feed-forward network motifs by which HeLa cells may maintain molecular homeostasis in transcriptional regulation.

**Figure 7:**
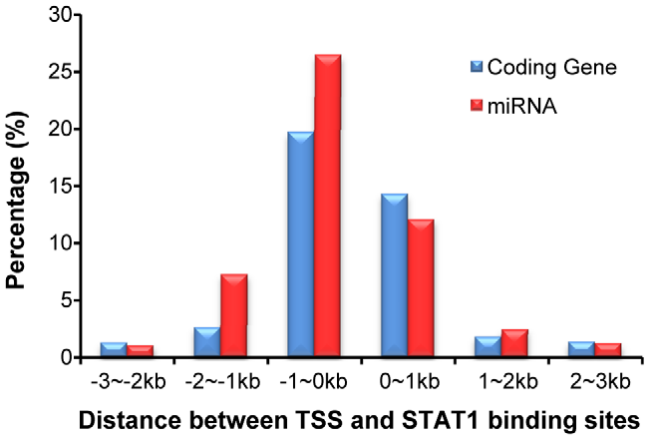
Percentage of genes containing STAT1 binding sites within every 1KB region surrounding transcription start sites. The calculation is based on 36,998 STAT1 binding sites identified in the PeakSeq algorithm with FDR≤0.05 [11] and their relative locations with 4,120 expressed coding genes and 83 predicted microRNAs. The genes with their binding sites beyond +/−3kb from TSS are not included in the plot.

The density of STAT1 binding sites in microRNA promoters is similar to the ones in protein- coding genes. We compared the density of STAT1 target sites related to the distance from transcription start sites, for both microRNAs and protein coding genes. For the 4,120 expressed coding genes and the 83 microRNAs predicted to be actively transcribed, we counted the number of STAT1 binding sites in every 1,000-bp interval from 3,000-bp upstream to 3,000-bp downstream of their transcription start sites. Among the genes that contain binding sites within these regions (−3,000bp to +3,000bp from TSS), the percentage of genes containing STAT1 targets in each 1,000-bp interval was calculated (Figure 7). We observed significant enrichment of STAT1 binding sites within −1,000 bp to +1,000 bp of the transcription start site, for both protein coding genes (34%) and microRNAs (38%). This suggests that STAT1 is involved in regulating primary microRNAs (pri-microRNAs) transcription, at a level similar to protein coding genes.

We have identified AP-1 as one of the collaborating transcription factors that preferably bind to the adjacent sites surrounding STAT1 binding regions. Several previous studies reported that JAK-STAT and AP-1 signaling pathways interact during interferon γ stimulation [31], [32], [33], [34]. Our study suggests that such interaction not only occurs in regulating individual genes; it is also important in regulating microRNA transcription. We observed that 51.4% of the STAT1-targeting microRNAs also contain AP-1 binding sites.

Eight STAT1-targeting microRNAs are potentially involved in the post-transcriptional regulation of at least one of the factors moderating interferon γ stimulation (Figure 5B). Of these microRNAs, miR-24-2 may target interferon-γ, FOS, and JUN, while miR-220c can potentially regulate interferon γ and STAT1. We checked the disease relevance of these two microRNAs using the miR2Disease database [35], a manually curated database that aims at documenting known relationships between microRNA dysregulation and human disease. The dysregulation of both these two microRNAs are reported to be related to multiple diseases, especially cancer. Expression abnormalities of both microRNAs are related to gliobalstoma [36], lung cancer [37], pancreatic cancer [38], [39], [40], and papillary thyroid carcinoma [41]. In addition, miR-220 is also reported to be deregulated in leukemia [42]; miR-24-2 is deregulated in colon cancer [38], stomach cancer [38], and liver cancer [43].

It is worth noting that the current motif analysis, including both binding sites of STAT1 and its collaborating factors, relies on the position specific scoring matrix (PSSM) in the TRANSFAC database [28]. *De novo* motif finding tools such as MEME [44] and MDScan [45] can be used for the transcription factors whose binding site consensuses are less well documented. More recently, a new computational algorithm, Hybrid Motif Sampler (HMS) was specifically designed for identifying *de novo* binding motifs from ChIP-seq-derived transcription factor binding data [46], which provides a powerful tool in understanding the sequence features of transcription factors from genome-wide binding data. Such analysis can also be integrated into our strategy to further decipher the microRNA-mediated regulatory network.

Similar to many other bioinformatics predictions, most conclusions derived from this study are speculative. The purpose of this study is to offer a bioinformatics strategy to provide testable hypotheses on the mechanisms of microRNA-mediated transcriptional regulation, based on genome-wide protein-DNA interaction data derived from ChIP-seq experiments. To test these hypotheses, experimental validation is necessary to completely understand the transcriptional and post-transcriptional mechanisms that regulate the global gene expression pattern.

## Materials and Methods

### microRNA promoter identification

We previously developed a computational strategy to predict the promoter regions of microRNAs based on the RPol II binding patterns around their transcription start sites [22]. Our model assumes that RPol II binding distribution around the TSS is similar for microRNAs and protein coding genes. Using maximum likelihood estimation, we identified the parameters that best described RPol II binding patterns around the TSS of highly expressed, well-annotated promoter regions of protein-coding genes, and we then scanned the upstream regions of all the intergenic microRNAs, searching for genomic regions statistically similar to RPol II binding patterns around the TSS of the coding genes. The features being used in the model include the intensity of RPol II ChIP-seq signal around the transcription start site, steady transcript region, steady background region, and the decay rates of the RPol II signal in the promoter and transcription regions, respectively [22].

### Binding sites of collaborating factors enriched in STAT1 binding regions

Position-specific score matrices (PSSM) were used to calculate the possibility of a specific transcription factor binding at one genomic locus, as described previously [29].

The STAT1 ChIP-enriched regions were scanned for transcription factor-binding motifs using 741 PSSM documented in the TRANSFAC database [28]. The highest matching score during this scanning was used to evaluate the binding potential of the candidate transcription factor in the specific STAT1 ChIP-enriched region. The background nucleotide sequences were selected from all of the 1,000-bp promoter sequences upstream of the transcription starting sites of known genes that are not overlapped with STAT1 ChIP-enriched region. We did not use a randomly selected intergenic region as background sequences to avoid the sequence bias caused by the unique features in the promoter regions, such as higher levels of GC content. For each PSSM, a unique cutoff score was determined by the lowest matching score, where the density of positively identified binding sites in the ChIP-enriched regions was five times more than the one in the background oligonucleotide sequences (or FDR of ≤20%). This analysis enables selecting transcription factors whose binding sites are enriched in ChIP-selected regions compared to background promoters. Similar strategies were used previously [47].

### microRNA target prediction

Without losing generalizability, we used TargetScan (version 5.0) for microRNA target prediction [48], [49]. TargetScan predicts biological targets of microRNAs by searching for the presence of conserved 8mer and 7mer sites that match the seed region of each microRNA. In this study, we used all the predicted human targets, which include all three types of seed sites conserved across mammals: 8mer (exact match to positions 2–8 of the mature microRNA followed by an ‘A’), 7mer-m8 (exact match to positions 2–8 of the mature microRNA), and 7mer-1A (exact match to positions 2–7 of the mature microRNA followed by an ‘A’).

## Supporting Information

Table S1 List of 83 microRNAs and their predicted transcription start sites and promoter regions
(0.04 MB XLS)

Table S2 Enriched transcription factor binding sites in STAT1 enriched regions
(0.03 MB XLS)

Table S3 Six transcription factors that potentially regulate STAT1-targeting microRNAs
(0.04 MB XLS)

Table S4 The List of STAT1-targeting genes that are also predicted targets of 37 STAT1-targeting microRNAs
(0.46 MB XLS)
